# Daily Use of Caloric and Artificial Sweeteners Among Hungarian Adults with Diabetes: Socioeconomic and Dietary Associations

**DOI:** 10.3390/nu18081279

**Published:** 2026-04-17

**Authors:** Battamir Ulambayar, Bene Ágnes, Marianna Móré, Attila Csaba Nagy

**Affiliations:** 1 Department of Epidemiology, Faculty of Health Sciences, University of Debrecen, 4032 Debrecen, Hungary; ulambayar.battamir@etk.unideb.hu; 2 Department of Gerontology, Institute of Social and Sociological Sciences, Faculty of Health Sciences, University of Debrecen, 4400 Nyiregyhaza, Hungary; bene.agnes@etk.unideb.hu (B.Á.); more.mariann@ek.unideb.hu (M.M.)

**Keywords:** diabetes mellitus, caloric sweeteners, artificial sweeteners, dietary behavior, socioeconomic factors, Hungary

## Abstract

***Background/Objectives:*** Dietary sweetener use is common among individuals with diabetes, yet little is known about the socioeconomic and dietary factors that influence the choice between caloric and artificial sweeteners in Central and Eastern Europe. This study examined the determinants of caloric and artificial sweetener use among adults with diabetes mellitus (DM) in Hungary. ***Methods:*** We conducted a cross-sectional analysis using frequency-based self-reported dietary measures of 542 adults with self-reported DM from the 2019 European Health Interview Survey (EHIS). Weighted descriptive statistics and multivariable logistic regression models were used to evaluate associations between sweetener use and sociodemographic, lifestyle, and dietary characteristics. ***Results:*** Overall, 28.0% of participants reported daily use of caloric sweeteners, and 45.2% reported daily use of artificial sweeteners. Higher educational attainment and healthier dietary patterns, including greater fruit and vegetable consumption, were associated with lower odds of caloric sweetener use. Individuals with lower fruit and vegetable intake, less frequent fruit juice consumption, and poor adherence to diabetic diet recommendations were more likely to use caloric sweeteners. In contrast, artificial sweetener use was strongly associated with daily fruit consumption, lower intake of fruit juice, and adherence to a diabetic diet. Vegetable intake showed a positive association with artificial sweetener use, which may reflect compensatory patterns in dietary self-management. ***Conclusions:*** Caloric and artificial sweetener use were associated with distinct socioeconomic and dietary profiles. Caloric sweetener use was linked to less healthy dietary behaviors, whereas artificial sweetener use appeared to be consistent with sugar-reduction strategies. These findings highlight the need for tailored dietary counseling that addresses both sweetener use and broader dietary patterns among individuals with diabetes in Hungary.

## 1. Introduction

Diabetes mellitus (DM) is a major global health challenge, affecting an estimated 537 million adults worldwide in 2021, with projections indicating continued growth driven by aging populations, lifestyle changes, and increasing obesity rates [1]. Hungary bears a particularly high burden: the prevalence of diagnosed diabetes has risen substantially over the past few decades, placing the country among those with the highest rates in the European Union [2,3]. Effective diabetes management requires sustained lifestyle modification, including adherence to dietary recommendations aimed at reducing caloric intake and controlling carbohydrate quality and quantity [4].

Sweetener use, both caloric and artificial sweeteners, plays a central role in dietary strategies for glycemic control. Artificial sweeteners are widely promoted as low- or zero-calorie alternatives that help reduce sugar intake and mitigate postprandial glucose responses [5,6]. However, debates persist regarding their long-term metabolic and cardiometabolic effects, including potential influences on insulin sensitivity, appetite regulation, and gut microbiota composition [7,8,9]. In contrast, some natural sweeteners are commonly perceived as ‘healthier’ alternatives; however, most of these, including table sugar and honey, contain similar amounts of simple carbohydrates and contribute comparably to glycemic load and energy intake [10]. Although some products, such as honey, contain trace bioactive compounds, their metabolic impact in terms of glucose regulation remains largely similar when consumed in typical dietary amounts [11]. This discrepancy between perceived and actual health effects is particularly relevant in the context of diabetes management and may reflect differences in health literacy related to sweetener choices. A recent meta-analysis of fructose-containing sugars (including honey or fructose–glucose mixtures) found that consumption of such sugars is associated with increased fasting blood glucose and elevated insulin levels in adults, compared with unsweetened controls [12].

Evidence increasingly shows that dietary behaviors, including sweetener choices, are not determined solely by clinical advice but are strongly shaped by socioeconomic status, cultural norms, taste preferences, and access to health information [13,14]. Lower socioeconomic position has consistently been associated with poorer diet quality, reduced adherence to dietary guidelines, and limited engagement in diabetes self-care behaviors [15,16]. Despite these insights, little is known about the socioeconomic and dietary determinants of sweetener use among individuals with DM in Central and Eastern Europe. In Hungary, data on sweetener consumption specifically within diabetic populations remain scarce, even though understanding these behaviors is crucial for informing culturally and socioeconomically tailored dietary counseling. Identifying differences between caloric and artificial sweetener users may provide valuable insights into dietary adherence, health literacy, and compensatory behaviors among people living with DM.

Therefore, the present study aims to explore the demographic, socioeconomic, and dietary factors associated with caloric and artificial sweetener use among adults with DM in Hungary. By characterizing distinct behavioral profiles associated with different sweetener types, this study contributes new evidence to support targeted public health and clinical nutrition interventions aimed at improving dietary management in patients with DM.

## 2. Materials and Methods

### 2.1. Study Design and Data

This cross-sectional study used data from the 2019 European Health Interview Survey (EHIS), a nationally representative survey conducted in Hungary as part of the third EHIS wave coordinated by Eurostat. The EHIS collects harmonized, population-based information on health status, health determinants, and healthcare use among individuals aged 15 years and older residing in private households across EU Member States. The Hungarian Central Statistical Office was responsible for survey implementation, applying a multistage, stratified probability sampling design to ensure representativeness by age, sex, and region. For the present analysis, we included adult respondents (≥18 years) who self-reported a diagnosis of DM. The initial pool consisted of all eligible respondents with self-reported diabetes in the Hungarian EHIS dataset. Participants with missing data on sweetener use or key covariates were excluded using a complete-case approach. After these exclusions, the final analytical sample comprised *N* = 542 adults with DM. Sampling weights provided by Eurostat were applied in all analyses to account for differential probabilities of selection and to produce nationally representative estimates.

### 2.2. Variables

Two primary outcome variables were examined based on standardized EHIS self-reported dietary items. Caloric sweetener use was defined as daily consumption of caloric sweeteners based on the EHIS questionnaire, which groups products such as added table sugar, honey, and other caloric sweeteners into a single category. It should be noted that this classification does not distinguish between different types of caloric sweeteners, despite potential differences in consumer perception and usage patterns. Furthermore, the EHIS questionnaire does not distinguish non-caloric natural sweeteners (e.g., stevia) from other non-nutritive sweeteners; therefore, these products could not be analyzed separately and are not included within the ‘caloric sweetener’ category. Artificial sweetener use refers to the daily consumption of non-nutritive sweeteners, including products containing aspartame, saccharin, or other low-calorie sweeteners. The EHIS questionnaire does not allow identification of specific products within each category nor the simultaneous use of multiple sweetener types. Therefore, individuals may be classified as users of both caloric and artificial sweeteners, and these categories are not mutually exclusive. Frequency responses were dichotomized into ‘daily’ versus ‘non-daily’ use to capture habitual consumption patterns and to ensure adequate statistical power across categories. More granular classification was not retained due to small cell sizes in intermediate frequency categories and the analytical focus on distinguishing regular versus occasional exposure.

A range of sociodemographic and health-related covariates was included based on prior literature and data availability. These variables comprised gender (male or female); age group (18–34, 35–64, or ≥65 years); educational attainment (primary, secondary, or higher education); and net equivalent household income, categorized into quintiles. Additional health-related measures included self-perceived health status (very good/good, satisfactory, or bad/very bad) and body mass index (BMI), classified as normal weight, overweight, or obese based on self-reported height and weight. Lifestyle factors such as smoking status (active, former, or never) and alcohol consumption (none, low-level, or moderate/heavy) were also included.

All dietary variables were assessed using frequency-based self-report measures. Although self-reported dietary data are subject to recall and social desirability bias, such instruments are widely employed in large-scale epidemiological surveys and are considered appropriate for ranking individuals according to the relative frequency of intake rather than estimating absolute quantities. In population-level analyses, this approach enables the identification of relative behavioral differences between subgroups while maintaining comparability across respondents. Original EHIS response categories for fruit, vegetable, and juice intake were recorded on an ordinal frequency scale (e.g., several times a day, once a day, 4–6 times a week, 1–3 times a week, less than once a week, never). For analytical purposes and to ensure adequate cell sizes, categories were collapsed into three groups: “every day”, “sometimes” (≥1 time per week but not daily), and “rarely/never” (<1 time per week or never). This categorization preserves ordinal structure while improving statistical stability. Missing data were handled using a complete-case approach. The proportion of missing values for the included variables was low, and therefore, imputation methods were not applied.

### 2.3. Statistical Analysis

All analyses incorporated EHIS survey weights to account for sampling design and ensure nationally representative findings. Descriptive statistics were used to summarize participant characteristics stratified by caloric and artificial sweetener use. Differences between users and non-users were evaluated using chi-squared tests for categorical variables. BMI was calculated from self-reported height and weight and is presented descriptively using boxplots stratified by sweetener use and gender to assess whether there are substantial differences in BMI between users and non-users of caloric or artificial sweeteners across gender groups. To identify factors independently associated with sweetener use, we conducted multivariable binary logistic regression analyses separately for caloric and artificial sweetener use. All models are fully adjusted models, including all covariates described above (age, gender, education, income, BMI, self-perceived health, smoking status, alcohol consumption, fruit intake, vegetable intake, fruit juice consumption, sweets/desserts intake, and adherence to a diabetic diet), selected a priori based on conceptual relevance and prior evidence. Potential multicollinearity among dietary variables (fruit, fruit juice, vegetable intake, sweets/desserts, and diabetic diet adherence) was evaluated using variance inflation factors (VIFs), all of which were below 2.5, indicating no significant collinearity. Interaction terms were not included in the models due to sample size considerations and to avoid model overfitting. Results are presented as odds ratios (ORs) with 95% confidence intervals (CIs). Statistical significance was defined as a two-sided *p* < 0.05. All analyses were conducted using STATA IC version 18.0 [17].

## 3. Results

**Figure 1:**
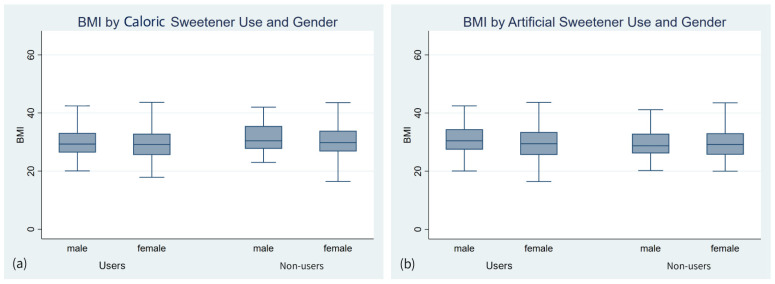
Distribution of BMI by caloric (**a**) and artificial sweetener (**b**) use and gender among adults with diabetes. Boxplots represent the median and interquartile range.

A total of 542 patients with DM were included in the study. Of these, 152 (28.0%) reported daily use of caloric sweeteners, and 245 (45.2%) reported daily use of artificial sweeteners (including aspartame and saccharin). Table 1 and Table 2 present the factors associated with the use of caloric and artificial sweeteners, respectively, among patients with DM. BMI distributions stratified by sweetener use and gender are shown in Figure 1. Visual inspection indicates no substantial differences in BMI between users and non-users of caloric or artificial sweeteners across gender groups.

In the bivariate analyses (Table 1), several characteristics were significantly associated with daily use of caloric sweeteners. Patients with higher educational attainment were less likely to report caloric sweetener use (*p* = 0.004). The prevalence was highest among those with only primary education (34.8%) and lowest among those with higher education (15.7%). Similarly, a clear inverse socioeconomic gradient was observed across household income quintiles (*p* = 0.045), with caloric sweetener use being most common in the lowest income quintile (36.6%) and progressively less frequent in higher quintiles (21.7% in the highest quintile). Daily fruit consumption was associated with lower use of caloric sweeteners (*p* = 0.021); 38.1% of those who consumed fruit only “sometimes” used caloric sweeteners compared with 25.9% of daily fruit consumers. Consumption of fruit juice showed a similar pattern (*p* = 0.025): daily consumers had the highest prevalence of caloric sweetener use (41.9%), whereas rare consumers had the lowest (24.5%). Frequent intake of sweets and desserts was strongly linked to caloric sweetener use (*p* = 0.002). Among patients consuming two or more portions of sweets/desserts per day, 36.3% used caloric sweeteners, compared with only 23.7% of those who never consumed such products. Not following a diabetic diet was one of the strongest correlates (*p* < 0.001): 35.2% of patients who did not adhere to a diabetic diet reported caloric sweetener use, versus only 21.7% of those who followed a diabetic diet. Alcohol consumption pattern also differed significantly (*p* = 0.019), with the lowest prevalence of caloric sweetener use observed among low-level drinkers (22.0%) and the highest among patients reporting no alcohol consumption (33.5%) or heavy/moderate use (32.4%).

Age was positively associated with artificial sweetener use (*p* = 0.047): prevalence increased from 31.6% in the youngest age group (18–34 years) to 40.1% in the 35–64-year-old group and 49.5% in those aged 65 years and older. Daily fruit consumption was associated with higher artificial sweetener use (*p* = 0.033): 49.0% of patients who ate fruit every day used artificial sweeteners, compared with 35.6% of those who consumed fruit only sometimes. Frequency of fruit juice consumption showed a strong inverse association (*p* = 0.003). Artificial sweetener use was most common among those who rarely consumed fruit juice (51.5%) and least common among daily fruit juice consumers (27.4%). Adherence to a diabetic diet was significantly related to artificial sweetener use (*p* = 0.004): 51.0% of patients who followed a diabetic diet reported using artificial sweeteners, versus 38.7% of those who did not follow such a diet (Table 2). However, some of these associations were attenuated or no longer statistically significant after adjustment for covariates in the multivariable models, indicating that unadjusted patterns should be interpreted with caution.

In the multivariable logistic regression analysis of factors associated with caloric sweetener use, several significant associations emerged, highlighting both lifestyle behaviors and socioeconomic factors (Table 3). Notably, individuals with higher education were significantly less likely to use caloric sweeteners compared to those with primary education (OR 0.41, 95% CI 0.19–0.89, *p* = 0.023), suggesting that education may shape dietary choices in nuanced ways, potentially reflecting greater awareness of sugar alternatives or differing health beliefs.

Dietary habits were strongly linked to sweetener use. Participants who consumed fruit occasionally had more than twice the odds of using caloric sweeteners compared to daily fruit consumers (OR 2.16, 95% CI 1.25–3.74, *p* = 0.006), while those with less frequent vegetable intake were less likely to use caloric sweeteners (OR 0.53, 95% CI 0.32–0.87, *p* = 0.012). Similarly, participants who rarely or never consumed fruit juice had lower odds of caloric sweetener use (OR 0.47, 95% CI 0.24–0.90, *p* = 0.023). These patterns may reflect broader dietary preferences and the interplay between caloric sweetener use and the overall consumption of fruits and vegetables.

Behavioral modifications related to health were also significant. Individuals not following a diabetic diet were more likely to use caloric sweeteners than those adhering to such a diet (OR 1.86, 95% CI 1.21–2.85, *p* = 0.005), which could be consistent with compensatory or preference-driven use of sweeteners outside structured dietary guidance. Conversely, low-level alcohol consumption was associated with reduced caloric sweetener use compared to moderate or heavy use (OR 0.54, 95% CI 0.30–0.97, *p* = 0.038), suggesting that different lifestyle patterns may cluster in ways that influence sweetener consumption.

In the multivariable logistic regression model examining factors associated with artificial sweetener use, dietary habits emerged as the most influential determinants (Table 4). Individuals who consumed fruit only sometimes were significantly less likely to use artificial sweeteners compared to daily fruit consumers (OR 0.50, 95% CI 0.30–0.83, *p* = 0.007), suggesting that regular fruit intake may align with broader patterns of sweetener use, potentially reflecting attention to sugar intake or taste preferences. Vegetable consumption was positively associated with the use of artificial sweeteners. Participants who consumed vegetables sometimes (OR 1.98, 95% CI 1.28–3.04, *p* = 0.002) or rarely/never (OR 2.44, 95% CI 1.11–5.38, *p* = 0.027) were more likely to use artificial sweeteners compared to daily vegetable consumers. Similarly, fruit juice intake showed a consistent trend: those consuming juice sometimes (OR 2.12, 95% CI 1.11–4.06, *p* = 0.023) or rarely/never (OR 2.59, 95% CI 1.35–4.96, *p* = 0.004) had higher odds of artificial sweetener use. These associations may reflect compensatory behaviors, where reduced intake of caloric sweet foods is balanced by the use of artificial alternatives. Patients who did not follow a diabetic diet were associated with lower artificial sweetener use compared to adherence to such a diet (OR 0.66, 95% CI 0.45–0.96, *p* = 0.032), emphasizing the role of health-focused dietary decisions in shaping sweetener consumption.

## 4. Discussion

This cross-sectional analysis aimed to identify socioeconomic and behavioral factors associated with the use of caloric and artificial sweeteners, both of which have been the subject of debate regarding their role in diabetes management. The results demonstrated that higher educational attainment and healthier dietary patterns, including greater fruit and vegetable consumption, were associated with lower odds of caloric sweetener use, whereas lower consumption of these foods was associated with higher odds of artificial sweetener use, as well as adherence to a diabetic diet.

The relationship between socioeconomic status and sweetener use is complex and reflects broader dietary inequalities. Research highlights the significant impact of socioeconomic status on dietary choices, including the consumption of sweeteners. Lower socioeconomic status individuals often have higher estimated consumption of sugar-sweetened beverages, which correlates with an increased prevalence of type 2 diabetes and metabolic syndrome within these demographics [18,19,20]. Specifically, studies indicate that lower-income groups consume greater amounts of sugar, potentially due to targeted marketing and accessibility issues associated with healthier alternatives [21], as shown in our results. These patterns may reinforce existing dietary inequalities, as individuals with lower socioeconomic status are more likely to rely on energy-dense, sugar-rich foods. However, recent research has shown that the consumption of artificial sweeteners is more likely driven by health considerations than by socioeconomic factors [8]. Our current study supports this finding, showing that socioeconomic factors such as income and education are not associated with artificial sweetener consumption.

The associations observed between fruit and vegetable intake and the use of caloric and artificial sweeteners suggest that these two groups of sweetener users represent distinct dietary profiles among adults with DM. Individuals who consumed fruits and vegetables less frequently demonstrated higher odds of caloric sweetener use. This pattern is consistent with previous evidence showing that lower fruit and vegetable intake is strongly associated with poorer overall diet quality, greater reliance on added sugars and energy-dense foods, and reduced engagement in health-promoting behaviors, including adherence to dietary guidelines for chronic disease management [22,23]. Habitual consumption of sugar-rich foods may also shape taste preferences, increasing the desire for added sweetness and reducing tolerance for naturally occurring sweetness in fruits. In this context, the lower fruit and vegetable consumption and poorer adherence to diabetic dietary recommendations observed among caloric sweetener users likely reflect broader suboptimal dietary behavior. Individuals with low exposure to the natural sweetness of whole fruits may rely more heavily on added sweeteners to achieve the desired taste [24]. This is further supported by evidence demonstrating that socioeconomic disparities are consistently linked to poorer dietary habits, including lower fruit and vegetable consumption among individuals from disadvantaged backgrounds [25].

An important consideration is the heterogeneity within the “caloric sweetener” category, which includes both added sugars and products such as honey. Although often perceived as healthier, these sweeteners have broadly similar effects on glycemic load and energy intake. From a metabolic perspective, caloric and artificial sweeteners differ substantially in their physiological effects, which has important implications for diabetes management. Caloric sweeteners, including sucrose-, glucose-, and fructose-containing products such as honey, directly contribute to energy intake and glycemic load, although their glycemic index may vary. While fructose-containing sweeteners are sometimes perceived as more favorable due to their lower glycemic index, they are primarily metabolized in the liver, where excessive intake may promote de novo lipogenesis, insulin resistance, and adverse metabolic outcomes. This distinction highlights that a lower glycemic index does not necessarily translate into improved metabolic health. In contrast, artificial sweeteners provide little or no energy and generally do not elicit an acute glycemic response, making them a useful tool for reducing sugar intake in the short term. However, growing evidence suggests that their long-term metabolic effects may be more complex, with potential influences on appetite regulation, glucose homeostasis, and gut microbiota composition, although findings remain inconsistent. Therefore, both caloric and artificial sweeteners present distinct advantages and limitations, and their metabolic effects should be interpreted within the broader context of overall dietary patterns and long-term health outcomes.

These metabolic differences may help to explain the behavioral patterns observed in our study. The higher prevalence of caloric sweetener use among individuals with poorer dietary habits may partly reflect misperceptions regarding the healthfulness of certain “natural” sweeteners, such as honey, which are often viewed as healthier alternatives despite having similar metabolic effects to refined sugar when consumed in typical amounts. This may lead to substitution without a meaningful reduction in total sugar intake [26,27]. In contrast, the use of artificial sweeteners was more common among individuals adhering to diabetic dietary recommendations and exhibiting patterns consistent with intentional sugar reduction, suggesting a more health-conscious or strategy-driven approach [28]. However, the coexistence of artificial sweetener use with suboptimal vegetable intake indicates that focusing solely on sugar reduction does not necessarily translate into overall diet quality.

It should also be noted that artificial sweeteners are a heterogeneous group of compounds, and emerging evidence suggests that different types may have varying effects on metabolic outcomes, including gut microbiota composition and insulin sensitivity [29]. While some studies indicate potential adverse effects, findings remain inconsistent and context-dependent. Due to the structure of the EHIS dataset, we were unable to distinguish between specific artificial sweeteners, which limits a more detailed interpretation of these potential differential effects.

The inverse association between vegetable intake and artificial sweetener use suggests a more targeted approach to dietary modification. Although artificial sweetener use was associated with adherence to a diabetic diet, the lower intake of vegetables indicates that changes may be focused primarily on sugar reduction rather than overall diet quality. One possible explanation is compensatory behavior, where individuals who restrict caloric sources of sweetness (fruit or fruit juice) rely more on artificial sweeteners to maintain sweetness in their diet [30]. Alternatively, this pattern may reflect selective adherence to dietary recommendations, prioritizing sugar avoidance over a balanced, guideline-driven diet. Differences in nutritional knowledge, food preferences, or access to dietary counseling may further contribute to this behavior.

These patterns may suggest that caloric sweetener users are more likely to follow a taste-driven or habitual dietary pattern characterized by lower fruit and vegetable intake, lower dietary adherence, and greater consumption of sugar-rich foods. In contrast, artificial sweetener use was associated with patterns that may reflect more intentional sugar-modifying behaviors, including reduced intake of fruit juice and the use of artificial sweeteners to limit caloric sugars. Although neither approach aligns perfectly with diabetes nutrition guidelines, which encourage whole fruit intake and recommend moderation with both caloric and artificial sweeteners, the outcomes highlight the need for tailored dietary counseling. Specifically, guidance should emphasize the health benefits of fruit and vegetable consumption, address misconceptions around “caloric” sweeteners, and clarify the appropriate, evidence-based role of artificial sweeteners within balanced diabetes self-management plans.

This study benefits from the use of nationally representative EHIS data, allowing for population-level insights into sweetener use among adults with DM in Hungary, and from the inclusion of a broad set of sociodemographic and dietary variables that enabled comprehensive multivariable analysis. However, several limitations should be noted. The cross-sectional design prevents causal inference. All dietary variables, including sweetener use, were based on self-report and therefore subject to recall bias and potential misclassification. Additionally, diabetes status was self-reported, and the EHIS dataset does not differentiate between diabetes types or treatment modalities such as insulin therapy. This represents an important limitation, as nutritional strategies, glycemic targets, and dietary behaviors may vary substantially depending on disease type and clinical management. The EHIS questionnaire assesses frequency rather than quantitative intake, limiting the precision of exposure measurement. Consequently, the findings should be interpreted as reflecting relative behavioral patterns rather than exact levels of sweetener consumption. Furthermore, unmeasured factors such as diabetes duration, nutritional knowledge, access to dietary counseling, and overall diet quality may have led to residual confounding. Finally, some subgroup sample sizes were modest, which may have reduced statistical power. Despite these limitations, the findings provide valuable evidence to inform more targeted dietary counseling strategies in this population.

## 5. Conclusions

In this nationally representative sample of Hungarian adults with DM, caloric and artificial sweetener use was associated with distinct socioeconomic and dietary profiles. Caloric sweetener use was associated with dietary patterns that were less aligned with recommended dietary behaviors, including lower fruit and vegetable intake and poorer adherence to diabetic diet recommendations, whereas artificial sweetener use was associated with patterns consistent with sugar-reduction strategies, reflected in higher daily fruit consumption, avoidance of fruit juice, and greater compliance with dietary guidance.

These findings highlight the complexity of sweetener-related behaviors in diabetes management and underline the need for tailored nutritional counseling that clarifies the appropriate role of both caloric and artificial sweeteners while emphasizing the importance of overall diet quality. For example, patients using caloric sweeteners such as honey may benefit from education on their comparable glycemic impact to sugar and guidance on reducing overall caloric sweetener intake. In contrast, patients using artificial sweeteners may require support in adopting a more balanced diet, particularly by increasing vegetable intake, rather than focusing solely on sugar reduction. Understanding these behavioral patterns may support more effective, individualized dietary interventions aimed at improving glycemic control and overall diet quality in this population.

## Figures and Tables

**Table 1:** Factors associated with caloric sweetener use among adults with DM.

Variables	Categories	Caloric Sweetener Use		*p*-Value *
		No (*n*, %)	Yes (*n*, %)	
Gender	Male	188 (74.0)	66 (26.0)	0.316
	Female	202 (70.1)	86 (29.9)	
Age group	18–34 years old	14 (73.7)	5 (26.3)	0.554
	35–64 years old	147 (69.3)	65 (30.1)	
	65 and older	229 (73.6)	82 (26.4)	
Education levels	Primary	88 (65.2)	47 (34.8)	**0.004**
	Secondary	216 (70.8)	89 (29.2)	
	High	86 (84.3)	16 (15.7)	
Quintiles based on net equivalent household income	First (lowest)	71 (63.4)	41 (36.6)	**0.045**
	Second	88 (67.2)	43 (32.8)	
	Third	87 (77.0)	26 (23.0)	
	Fourth	97 (76.0)	29 (23.0)	
	Fifth (highest)	47 (78.3)	13 (21.7)	
Self-perceived health status	Very good or good	88 (75.8)	28 (24.2)	0.125
	Satisfactory	205 (73.5)	74 (26.5)	
	Very bad or bad	95 (65.5)	50 (34.5)	
BMI	Normal	70 (75.3)	23 (24.7)	0.369
	Overweight	144 (74.2)	50 (25.8)	
	Obese	170 (69.1)	76 (30.9)	
Fruit consumption per week	Everyday	286 (74.1)	100 (25.9)	**0.021**
	Sometimes	73 (61.9)	45 (38.1)	
	Rarely or never	27 (79.4)	7 (20.6)	
Vegetable consumption per week	Everyday	194 (70.0)	83 (30.0)	0.641
	Sometimes	161 (73.8)	57 (26.2)	
	Rarely or never	29 (72.5)	11 (27.5)	
Fruit juice consumption (no sugar or no artificial sweetener)	Everyday	36 (58.1)	26 (41.9)	**0.025**
	Sometimes	165 (71.7)	65 (28.3)	
	Rarely or never	179 (75.5)	58 (24.5)	
Servings of sweets and desserts a day	Two or more portions	58 (63.7)	33 (36.3)	**0.002**
	Once or less	50 (60.2)	33 (39.8)	
	Never	277 (76.3)	86 (23.7)	
Follows a diabetic diet	Yes	224 (78.3)	62 (21.7)	**<0.001**
	No	166 (64.8)	90 (35.2)	
Alcohol consumption	Heavy or moderate use	71 (67.6)	34 (32.4)	**0.019**
	Low-level use	174 (78.0)	49 (22.0)	
	No alcohol use	137 (66.5)	69 (33.5)	
Smoking status	Active smoking	72 (69.9)	31 (30.1)	0.239
	Former smoking	120 (76.9)	36 (23.1)	
	Never smoking	190 (69.6)	83 (30.4)	

* Bold values indicate statistical significance (*p* < 0.05) based on Pearson’s chi-squared test.

**Table 2:** Factors associated with artificial sweetener use among adults with DM.

Variables	Categories	Artificial Sweetener Use		*p*-Value *
		No (*n*, %)	Yes (*n*, %)	
Gender	Male	139 (54.7)	115 (45.3)	0.975
	Female	158 (54.9)	130 (45.1)	
Age group	18–34 years old	13 (68.4)	6 (31.6)	**0.047**
	35–64 years old	127 (59.9)	85 (40.1)	
	65 and older	157 (50.5)	154 (49.5)	
Education levels	Primary	71 (52.6)	64 (47.4)	0.835
	Secondary	169 (55.4)	136 (44.6)	
	High	57 (55.9)	45 (44.7)	
Quintiles based on net equivalent household income	First (lowest)	60 (53.6)	52 (46.4)	0.448
	Second	76 (58.0)	55 (42.0)	
	Third	55 (48.7)	58 (51.3)	
	Fourth	75 (59.5)	51 (40.5)	
	Fifth (highest)	31 (51.7)	29 (48.3)	
Self-perceived health status	Very good or good	68 (58.6)	48 (41.4)	0.166
	Satisfactory	142 (50.9)	137 (49.1)	
	Very bad or bad	86 (59.3)	59 (40.7)	
BMI	Normal	49 (52.7)	44 (47.3)	0.224
	Overweight	97 (50.0)	97 (50.0)	
	Obese	143 (58.1)	103 (41.9)	
Fruit consumption per week	Everyday	197 (51.0)	189 (49.0)	**0.033**
	Sometimes	76 (64.4)	42 (35.6)	
	Rarely or never	20 (58.8)	14 (41.2)	
Vegetable consumption per week	Everyday	159 (57.4)	118 (42.6)	0.298
	Sometimes	110 (50.5)	108 (49.5)	
	Rarely or never	21 (52.5)	19 (47.5)	
Fruit juice consumption	Everyday	45 (72.6)	17 (27.4)	**0.003**
	Sometimes	126 (54.8)	104 (45.2)	
	Rarely or never	115 (48.5)	122 (51.5)	
Servings of sweets and desserts a day	Two or more portions	52 (57.1)	39 (42.9)	0.482
	Once or less	49 (59.0)	34 (41.0)	
	Never	191 (52.6)	172 (47.4)	
Follows a diabetic diet	Yes	140 (49.0)	146 (51.0)	**0.004**
	No	157 (61.3)	99 (38.7)	
Alcohol consumption	Heavy or moderate use	66 (62.8)	39 (37.2)	0.100
	Low-level use	112 (50.2)	111 (49.8)	
	No alcohol use	113 (54.8)	93 (45.2)	
Smoking status	Active smoking	61 (59.2)	42 (40.8)	0.398
	Former smoking	79 (50.6)	77 (49.4)	
	Never smoking	147 (53.8)	126 (46.2)	

* Bold values indicate statistical significance (*p* < 0.05) based on Pearson’s chi-squared test.

**Table 3:** Multivariable logistic regression analysis of factors associated with caloric sweetener use.

Variable	Category	Odds Ratio	95% CI	*p*-Value *
Age group	18–34 years (ref)			
	35–64 years	1.17	0.33–4.13	0.802
	≥65 years	1.12	0.32–3.91	0.865
Education	Primary education (Ref)			
	Secondary education	1.00	0.60–1.67	0.991
	Higher education	0.41	0.19–0.89	**0.023**
Household income	First quintile (Ref)			
	Second	0.78	0.42–1.42	0.410
	Third quintile	0.53	0.27–1.03	0.060
	Fourth quintile	0.61	0.31–1.19	0.146
	Highest quintile	0.71	0.30–1.68	0.433
Self-perceived health status	Very good/good (Ref)			
	Satisfactory	0.98	0.56–1.74	0.952
	Bad/very bad	1.40	0.73–2.67	0.313
BMI	Normal (Ref)			
	Overweight	0.96	0.51–1.82	0.902
	Obese	1.12	0.60–2.10	0.713
Fruit consumption	Every day (Ref)			
	Sometimes	2.16	1.25–3.74	**0.006**
	Rarely or never	0.82	0.30–2.24	0.693
Vegetable consumption	Every day (Ref)			
	Sometimes	0.53	0.32–0.87	**0.012**
	Rarely or never	0.52	0.22–1.27	0.155
Fruit juice consumption	Every day (Ref)			
	Sometimes	0.60	0.31–1.14	0.117
	Rarely or never	0.47	0.24–0.90	**0.023**
Servings of sweets and desserts a day	Two or more portions			
	Once or less	1.28	0.64–2.54	0.487
	Never	0.64	0.37–1.12	0.120
Follows a diabetic diet	Yes (Ref)			
	No	1.86	1.21–2.85	**0.005**
Alcohol consumption	Heavy or moderate use (Ref)			
	Low-level use	0.54	0.30–0.97	**0.038**
	No alcohol use	0.87	0.49–1.54	0.630

* Bold values indicate statistical significance (*p* < 0.05). Odds ratios (ORs) are adjusted for sociodemographic, lifestyle, and dietary variables (age, gender, education, income, BMI, self-perceived health, smoking, alcohol consumption, and dietary factors) in the binary logistic regression model.

**Table 4:** Multivariable logistic regression analysis of factors associated with artificial sweetener use.

Variable	Category	Odds Ratio	95% CI	*p*-Value *
Age group	18–34 years (ref)			
	35–64 years	1.57	0.49–5.04	0.445
	≥65 years	2.06	0.65–6.54	0.220
Education	Primary education (Ref)			
	Secondary education	0.93	0.58–1.50	0.771
	Higher education	1.19	0.62–2.26	0.599
Household income	First quintile (Ref)			
	Second	0.78	0.44–1.38	0.390
	Third quintile	1.32	0.73–2.39	0.365
	Fourth quintile	0.75	0.41–1.37	0.348
	Highest quintile	0.98	0.46–2.09	0.955
Self-perceived health status	Very good/good (Ref)			
	Satisfactory	1.35	0.83–2.19	0.231
	Bad/very bad	0.89	0.50–1.59	0.704
BMI	Normal (Ref)			
	Overweight	1.17	0.68–2.01	0.580
	Obese	0.92	0.53–1.58	0.756
Fruit consumption	Every day (Ref)			
	Sometimes	0.50	0.30–0.83	**0.007**
	Rarely or never	0.61	0.26–1.43	0.254
Vegetable consumption	Every day (Ref)			
	Sometimes	1.98	1.28–3.04	**0.002**
	Rarely or never	2.44	1.11–5.38	**0.027**
Fruit juice consumption	Every day (Ref)			
	Sometimes	2.12	1.11–4.06	**0.023**
	Rarely or never	2.59	1.35–4.96	**0.004**
Servings of sweets and desserts a day	Two or more portions			
	Once or less	0.95	0.49–1.82	0.868
	Never	1.07	0.64–1.79	0.797
Follows a diabetic diet	Yes (Ref)			
	No	0.66	0.45–0.96	**0.032**
Alcohol consumption	Heavy or moderate use (Ref)			
	Low-level use	1.56	0.93–2.63	0.094
	No alcohol use	1.32	0.77–2.27	0.311

* Bold values indicate statistical significance (*p* < 0.05). Odds ratios (ORs) are adjusted for sociodemographic, lifestyle, and dietary variables (age, gender, education, income, BMI, self-perceived health, smoking, alcohol consumption, and dietary factors) in the binary logistic regression model.

## Data Availability

The data analyzed in this study are subject to the following licenses/restrictions: The data presented in this study are available upon request from the Hungarian Central Statistical Office, whose staff performed and supervised the data collection. Requests to access these datasets should be directed to the Hungarian Central Statistical Office, www.ksh.hu/?lang=en (accessed on 18 March 2026).
